# Deep Learning for Understanding Satellite Imagery: An Experimental Survey

**DOI:** 10.3389/frai.2020.534696

**Published:** 2020-11-16

**Authors:** Sharada Prasanna Mohanty, Jakub Czakon, Kamil A. Kaczmarek, Andrzej Pyskir, Piotr Tarasiewicz, Saket Kunwar, Janick Rohrbach, Dave Luo, Manjunath Prasad, Sascha Fleer, Jan Philip Göpfert, Akshat Tandon, Guillaume Mollard, Nikhil Rayaprolu, Marcel Salathe, Malte Schilling

**Affiliations:** ^1^ Ecole Polytechnique Fédérale de Lausanne, Lausanne, Switzerland; ^2^ neptune.ml, Warsaw, Poland; ^3^ Warsaw University of Technology, Warsaw, Poland; ^4^ deepsense.ai, Warsaw, Poland; ^5^ Centre for Natural Resources Management, Analysis, Training and Policy Research (NARMA), Kathmandu, Nepal; ^6^ Zurich University of Applied Sciences, Zürich, Switzerland; ^7^ Anthropocene Labs, New York, NY, United States; ^8^ Center for Cognitive Interaction Technology (CITEC), Bielefeld University, Bielefeld, Germany; ^9^ International Institute of Information Technology Hyderabad, Hyderabad, India

**Keywords:** deep learning, machine learning, remote sensing, satellite imagery, semantic segmentation

## Abstract

Translating satellite imagery into maps requires intensive effort and time, especially leading to inaccurate maps of the affected regions during disaster and conflict. The combination of availability of recent datasets and advances in computer vision made through deep learning paved the way toward automated satellite image translation. To facilitate research in this direction, we introduce the Satellite Imagery Competition using a modified SpaceNet dataset. Participants had to come up with different segmentation models to detect positions of buildings on satellite images. In this work, we present five approaches based on improvements of U-Net and Mask R-Convolutional Neuronal Networks models, coupled with unique training adaptations using boosting algorithms, morphological filter, Conditional Random Fields and custom losses. The good results—as high as AP=0.937 and AR=0.959—from these models demonstrate the feasibility of Deep Learning in automated satellite image annotation.

## 1. Introduction

Despite substantial advances in global human well-being, the world continues to experience humanitarian crizes and natural disasters. Long-term and reignited conflicts affect people in many parts of the world, but often, accurate maps of the affected regions either do not exist or are outdated by disaster or conflict. Satellite imagery is readily available to humanitarian organizations, but translating images into maps is an intensive effort. Today, maps are produced by specialized organizations or in volunteer events such as mapathons, where imagery is annotated with roads, buildings, farms, rivers etc. In this work, we explore how machine learning can help pave the way for automated analysis of satellite imagery to generate relevant and real-time maps.

Applications of the state-of-the-art results in deep learning have been increasingly accessible to various different domains over the last few years (LeCun et al., 2015), the main reasons being the advent of end-to-end approaches in deep learning (LeCun et al., 2015), and the access to vast amounts of openly available data and high performance compute. The same does however not hold true for the research community interested in satellite imagery and remote sensing. While access to high-performance compute infrastructure has not been an inhibiting factor, access to high-resolution imagery still stays a major inhibiting factor to high quality AI/ML research in satellite imagery and remote sensing.

This work builds on top of a recently released open dataset, SpaceNet (*v*1) (Spacenet on aws, 2018), which in partnership with Digital Globe, released raw multiband satellite imagery of (as high as) 30 cm resolution for numerous cities like Vegas, Paris, Shanghai, Khartoum, along with the corresponding annotations of buildings and roads. In this work, we focus on the problem of instance segmentation on a simplified version of the SpaceNet dataset, in order to detect buildings in different urban settings on high resolution satellite imagery. A large-scale competition was organized by the challenge platform crowdAI, which released a simplified version (details in Section 3) of the SpaceNet dataset, and attracted 55 participants and 719 submissions. In general, different architectures for image segmentation have been proposed in the past. Mask R-Convolutional Neuronal Networks (CNN) and U-Net type of architectures are currently seen as state-of-the-art for such problems. This has been further substantiated by the success of such architectures in this competition and as well for the application in satellite imagery. The top contestants all fall into these two basic categories and both show that they compete on a similar high level. Five different adaptations of U-Net and Mask-RCNN based approaches were applied in context of this problem and showed top performance in the segmentation challenge. The different improvements and results for these five approaches are outlined in this paper. The next section will review related work with a particular focus on the development of U-Net and Mask-RCNN types of architectures. This will be followed by a brief description of the used dataset and the applied evaluation metrics. Afterwards, the different methods will be explained and presented together with accompanying results and we will analyze the effect of the depth of the U-Net structure on results. A brief section will provide a comparison of the approaches followed by the conclusion.

## 2. Related Work

Semantic segmentation deals with the task of assigning each pixel in a given image to one of potentially multiple classes. It deals with recognizing which objects are shown and where exactly these are presented in the image. As such, it is a challenging task that requires, on the one hand, to take into account the overall context of the image and for each pixel that of the surrounding area. On the other hand, it is required to label each pixel individually focusing on a very fine level of detail. While approaches to semantic segmentation have been around for a long time (see review on more traditional approaches in Thoma (2016), or for example, He et al. (2004), Shotton et al. (2009)), the recent success of Deep Neural Networks in image related tasks (Krizhevsky et al., 2012) has translated as well to the area of semantic segmentation. Deep Neural Networks and in particular Convolutional Neuronal Networks have revolutionized the area of image classification during the last decade and are now the dominant approach for image classification leading to deeper and deeper architectures (He et al., 2016). This became possible through algorithmic advances—as using rectified-linear units that avoid vanishing of the gradient during training (Krizhevsky et al., 2012)—, as well as implementing convolutional and pooling layers that had originally been proposed long before (Fukushima, 1980). Such approaches deal with the question of what is shown in a given image. Using convolutional filters—that only focus on small portions of the image and are moved over the whole image—allows to learn subsequently more and more abstract structures and invariances in images. Learning becomes efficient through weight sharing and the whole network can be trained in an end-to-end fashion. Together with pooling layers, the focus and receptive field of each deeper layer successively broadens until a very coarse latent space summarizes input from large portions of the image and can be used for classification. This step-by-step abstraction helps to resolve invariances as translations of objects and supports classification. A drawback, however, is that this abstraction looses resolution and fine details of structure as needed in semantic segmentation.

While classification addresses what is shown in an image, semantic segmentation in addition deals with where exactly something is shown in the image. None-the-less, the introduction of Deep Learning techniques into semantic segmentation improved dramatically segmentation accuracy and therefore became the predominant approach in this area as well. This further promoted the area and the increasingly better results lead to broad application of approaches in commercial products.

In the following, we will review prominent developments on semantic segmentation using Deep Neural Networks. In particular, the focus will be on U-Net like approaches employing forms of convolutions together with deconvolution or upsampling as well as Mask R-CNN because in the described challenge these kinds of approaches showed to be the best performing ones. For a broader overview: There are different surveys and reviews on the current state of semantic segmentation. Lateef and Ruichek (2019) provide a systematic and exhaustive review of different categories of approaches employing Deep Learning techniques and presenting available benchmarks and datasets as well as evaluation criteria. Hao et al. (2020) put a different focus on the degree of supervision during training. More traditional approaches are summarized in Thoma (2016) and recent advances are briefly addressed in Atif et al. (2019) and Minaee et al. (2020).

A crucial first architecture was given by the Fully Convolution Network (FCN) (Long et al., 2015) that can be applied to images of any dimension. In general, it is using a convolutional network architecture for the first layers: blocks of convolution and max pooling layers are applied in sequence until the image is downsized to 1/32th of the original dimensions. While in classification afterward fully-connected layers would be utilized on this latent space, in FCNs class predictions are made on this level of detail for the different small clusters. Afterwards, the assigned labels are scaled up to its original size using a sequence of up sampling and deconvolutional layers. While the down stream is collecting contextual information in larger areas and for a coarse resolution, the up stream is tasked with reconstructing more detailed spatial information. This architecture lead to nice improvements on the PASCAL VOC dataset in 2012 and has in particular the advantage that it can be trained in an end-to-end fashion without requiring selection of features or tuning of these. Architectures of such a type employing convolutional layers are today the standard approach for semantic segmentation tasks (Lateef and Ruichek, 2019).

One disadvantage, that was found in the early approaches using DNNs for semantic segmentation, is that detailed structure tends to get lost and fine structures in images appear washed-out. FCN addressed this, on the one hand, by not using a very deep architecture, which would otherwise lead to overly large receptive fields, and, on the other hand, for the last steps they already introduced skip connections. Skip connections provide information from earlier layers in the processing sequence that operate on a more fine grained resolution. The outputs of these previous layers are used as an additional input to the later stage that is not only getting information from the directly preceding layer, but as well as the rerouted information from an earlier layer through the skip connections. The weights of these connections are adapted during training as well. This idea of skip connections has been further refined in U-Net type architectures and has in general be found to be quite effective (e.g., see Chen et al., 2018) which used short cut connections that enhanced the results). U-Net (Ronneberger et al., 2015) is an improvement of FCN and constituted of a symmetric arrangement of a contractive and an expansive path. Following a general trend toward smaller convolutional filters, the contractive path consists of a sequence of two 3×3 convolutions that is followed by a two-by-two max pooling layer. The expansive path is symmetric, but up-convolutions replace the max pooling layers. Importantly, corresponding layers of both paths are connected by skip connections (see Figure 5). These provide detailed information for the upscaling layer that has the same resolution as required for the output of that layer in the expansive path. U-Net provides a simple architecture that has become very popular as it can be implemented quite efficiently and the introduction of local information on every level of detail lead to much improved results. Many further architectures were built following a general U-Net or encoder-decoder like structure. For example, exchanging the different blocks of processing (convolutions and pooling layers) with refined and further improved blocks. Drozdzal et al. (2016) introduced residual blocks that added further skip connections inside each block as residual connections. This, in general, allows for deeper networks and better training which showed in their results as well. As a further step, Jégou et al. (2017) applied two dense blocks in each of the streams that both consist of multiple stacked layers—of convolutions—that are connected by residual connections and the information from all the layers inside that block is aggregated through skip-like connections at the output of a block. As a result, the output of each block contains low level as well as high level features at different resolution. This provided further state-of-the-art results.

In general, downsampling in the down stream—the concatenation of convolutions and pooling operation—aims at increasing the receptive field and taking more context into account as required for classification. But this increase comes with a reduced spatial resolution. As an alternative, dilated convolutions (Yu and Koltun, 2015) as well increase the receptive field without reducing spatial resolution. In dilated (or atrous) convolutions, a convolutional filter is build, but in this case the subsequent entries of the filter are not applied to subsequent entries in the input, but only every *l*-th entry of the input is processed with *l* being the dilation factor that represents space between entries in a filter. This increases the size of the receptive field dramatically (over multiple layers it increases exponentially) and still can be implemented reasonably efficient as sparse convolutions. As a result, such layers allow to derive contextual information at multiple scales without losing resolution (Yu and Koltun, 2015). Already the initial approach showed state-of-the-art performance. As one disadvantage, dilated convolutions tend to produce gridding artifacts that stem from the systematic structure of the constructed filters. Further improvements used spatial pyramid pooling modules, as for example in DeepLab (Chen et al., 2018a) or DeepLabv3+ in which this is complemented by a simple decoder module (Chen et al., 2018b). As an alternative, recently, FastFCN was developed (Wu et al., 2019). As processing dilated convolutions requires quite some memory and time, this approach started from FCN including recent improvements, but ultimately replaced dilated convolutions in the expansive path by Joint Pyramid Upsampling. This showed to be more efficient and still produced good results.

While one advantage of the earlier proposed deep architectures was the possibility to train these in an end-to-end fashion, other approaches used additional pre- and post-processing. DeepLab (Chen et al., 2018a) applied Conditional Random Fields (CRF) (Krähenbühl and Koltun, 2012) in a post-processing step which in their case produced better outlines of objects in semantic segmentation. CRFs had been applied in the past as a post-processing step that takes contextual information nicely into account and leads to more coherent labels. CRFs have been tested in one approach in the here described challenge, but it was found that such an explicit step can become unnecessary and appeared not helpful when sufficient detail was already trained into the Deep Neural Network (which is in agreement with other findings). Others have successfully integrated CRFs (Zheng et al., 2015) into training of the whole system as these tend to produce quite good results close to object boundaries.

Regional proposal based methods follow a different type of approach that has shown success in the past as well as in the results presented in this paper. Faster R-CNN (Ren et al., 2015) and Mask R-CNN (He et al., 2017) are examples of this type of architecture. These kind of approaches consist of multiple stages. First, regions of the input image are identified and bounding boxes for possible objects are proposed. In Faster R-CNN (Ren et al., 2015) a region proposal network was introduced as a form of a fully convolutional network. Secondly, features are extracted for each of these bounding boxes. In Faster R-CNN this was realized quite efficiently as both stages can share features that are detected using convolutional layers. In Mask R-CNN a third step is applied, in which—in the same way as in a FCN—the extracted features are not used for classification for the object in that bounding box, but instead are used for detailed pixel-wise prediction of class labels.

For a more detailed overview see Lateef and Ruichek (2019) and Minaee et al. (2020).

## 3. Dataset

**FIGURE 1:**
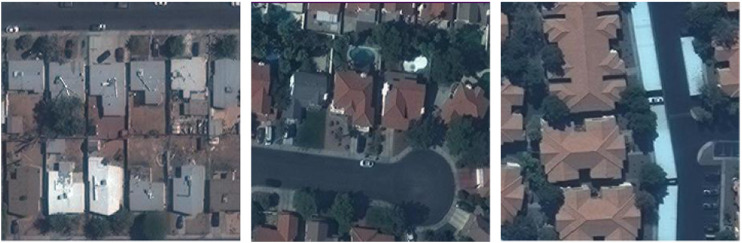
Sample images from the Mapping Challenge Dataset showing the top-down view of satellite imagery.

The dataset used in this work was derived from the SpaceNet dataset (Spacenet on aws, 2018). It provides a good dataset for comparing learning approaches on remote sensing data (for a comparable dataset see Castillo-Navarro et al. (2019)). Instead of considering all the channels in the multiband imagery from the SpaceNet dataset, we only focus on the RGB channels (for an example of an approach exploiting as well spectral information see Ben Hamida et al. (2017)). The decision to exclude information from non-RGB channels helps create an alternate version of the SpaceNet dataset, which makes the problem easy and accessible to researchers in Deep Learning, who may or may not be very familiar with the tools used by the Remote Sensing community to manipulate the multiband imagery, and are usually more familiar with simple RGB images which are extensively utilized in Deep Learning research. Moreover, when considering only the RGB channels, the problem becomes a direct parallel of very popular instance segmentation tasks commonly studied in Deep Learning research. At the same time, given the flexibility of most of the approaches in Deep Learning, if we demonstrate that we can get good results using just the RGB channels, extending the same approach to a multi channel signal provides us with better results. The dataset consists of a training set of 280,741 images, validation set of 60,317 images and test set of 60,697 images.^a^ (See Fig. 1)

## 4. Evaluation Metrics

The evaluation was principally based on the Intersection of Union (IoU) between the predicted mask and the ground truth.

For a known ground truth mask *A*, a predicted mask *B*, we first compute IoU (Intersection Over Union):

$$IoU\left(A,B\right)=\frac{A\cap B}{A\cup B}$$

IoU measures the overall overlap between the true region and the proposed region.

Then we consider a *True* detection, when there is at least half an overlap (or IoU≥0.5).

We can then define the following parameters (TP - true positive prediction, FP - false positive, FN - false negative):

- Precision (IoU≥0.5)
  $$P_{IoU\geq 0.5}=\frac{TP_{IoU\geq 0.5}}{TP_{IoU\geq 0.5}+FP_{IoU\geq 0.5}}$$
- Recall (IoU≥0.5)
  $$R_{IoU\geq 0.5}=\frac{TP_{IoU\geq 0.5}}{TP_{IoU\geq 0.5}+FN_{IoU\geq 0.5}}$$

The final scoring parameters

$$AP_{IoU\geq 0.5}$$

(average precision) and

$$AR_{IoU\geq 0.5}$$

(average recall) are computed by averaging over all the precision and recall values for all known annotations in the ground truth.

**FIGURE 2:**
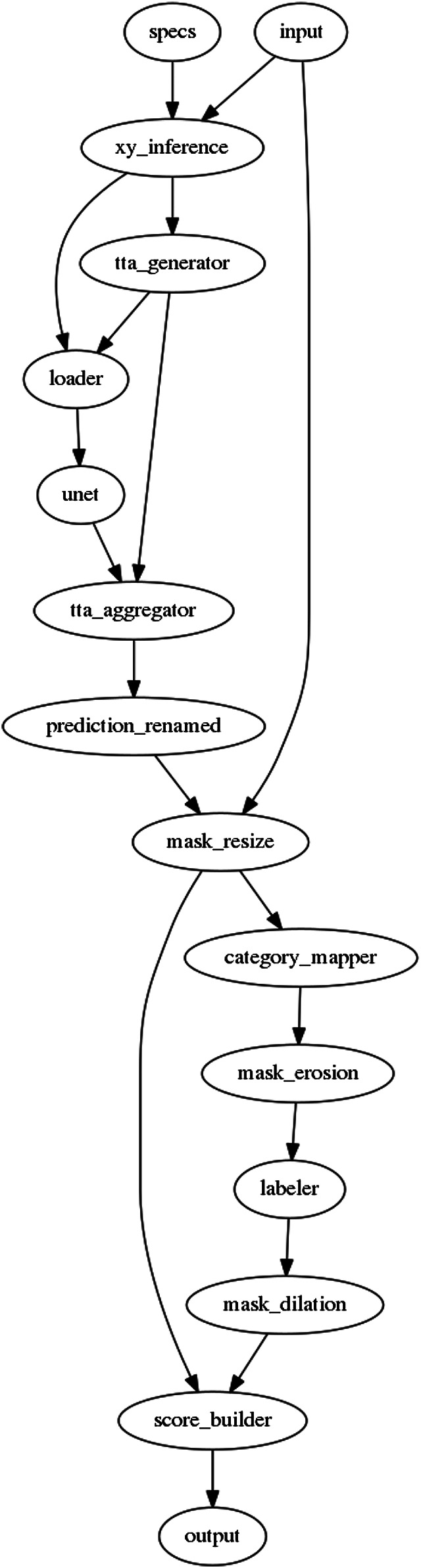
Schematic overview of the U-Net pipeline used in the first approach (Section 5). Nodes denote computational steps, arrows denote data flow. Please refer to **Table 1** for description of each node.

**FIGURE 3:**
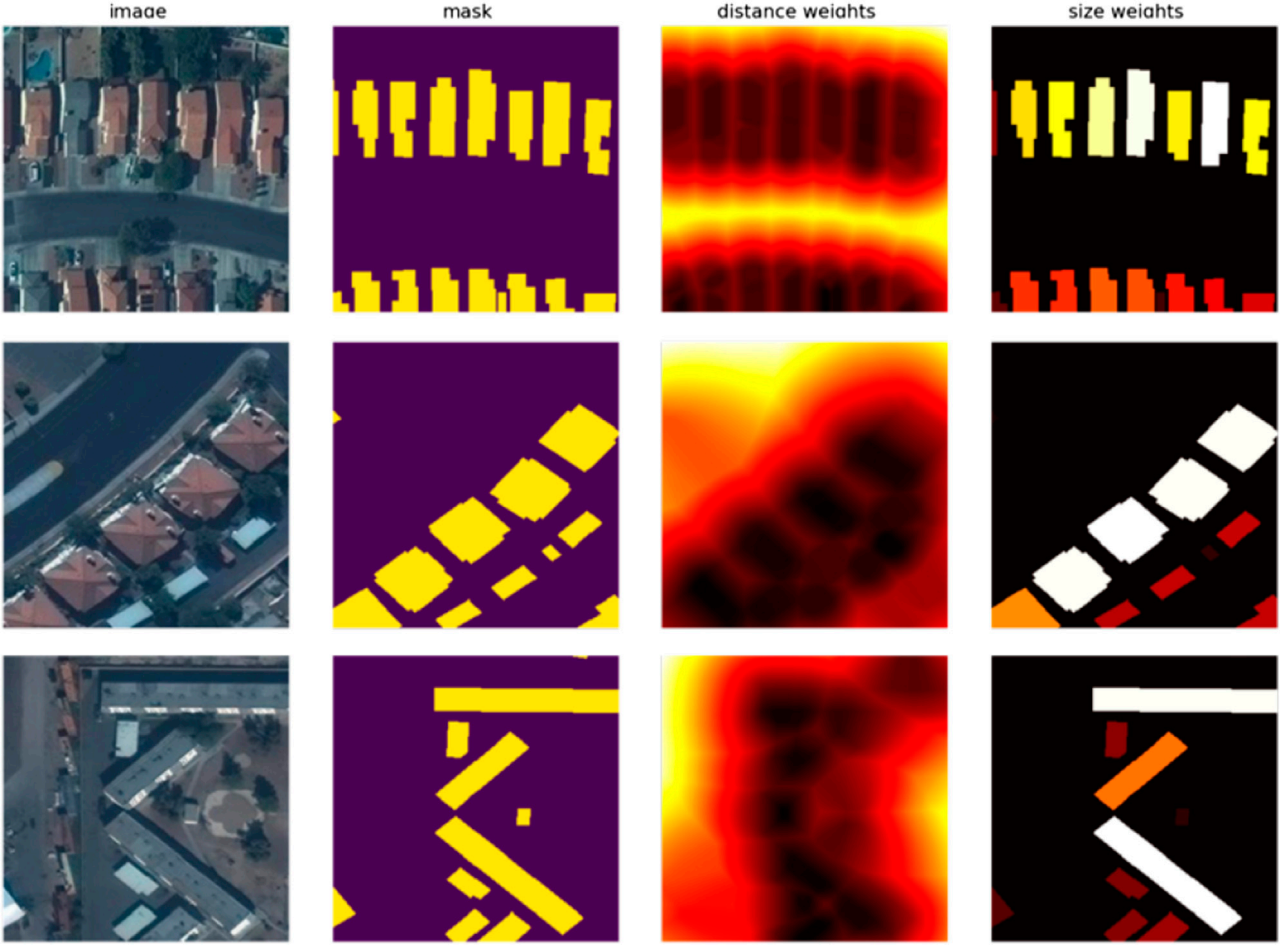
Each row represents a randomly sampled tile from the dataset. The 1^st^ column shows the input RGB image. The 2^nd^ column shows the ground truth mask. The 3^rd^ column visualizes the pixel-wise weight computed from the (inverse of) distance of a pixel to the two nearest buildings; high values corresponds to pixels between nearby buildings. The 4^th^ column visualizes the pixel-wize weight computed from the (inverse of) size of an instance; high values denote small buildings (the smaller the building the darker the color). Note, that the background is fixed to black for both the weight component visualizations.

**TABLE 1:** Experiment results for the U-Net adaptation using Fast Learnings for Fast Mapping (Section 6).

	No. of epochs	Training time (h)	Accuracy	Dice score	Average precision	Average recall
1 cycle only	20	38	99.22	98.25	91.74	92.19
1 cycle and cyclical learning rate × 2	40	76	99.36	98.56	91.76	92.86

## 5. Instance Segmentation Using Customized U-Net

As a first approach, we propose a two stage solution. The first stage is a neural network stage based on a U-Net (Ronneberger et al., 2015) followed by a post-processing stage using gradient boosting (Ke et al., 2017). Figure 2 presents the entire U-Net based pipeline, which takes raw RGB images along with some previously calculated meta-data as input, and predicts the instance segmentation masks.

1. Customized U-Net based Neural Network: Inspired by Iglovikov et al. 2018, we experimented with U-Net with Resnet34, Resnet101 (He et al., 2016) and Resnet152 as an encoder, and the best results were obtained in the case of Resnet101.
2. Loss Design: From the initial experiments, we quickly recognized the importance of closely placed buildings, and the tendency of the initial networks to group closely placed buildings as a single instance. The models also struggled with small instances (buildings with a small area in the image), but are equally important for the final evaluation metric (as the metric treats all instances equally when computing the Average Precision and Recall). There were many such cases, where an instance annotation was represented by barely a few pixels, as these were annotations from a building which was overflowing from the adjacent tile, and had only a small part of the building visible (and annotated) in the current tile. In order to be robust to these issues, we have designed a custom loss function by introducing two weighting factors (see Figure 3 for the visualization of weighting factors). The first factor puts a higher weight on pixels that are close to other buildings, and second factor puts a higher weight to pixels that belong to small objects. Equation 1 represents the loss function used in this approach.
  Let:
  - *x* predictions from U-Net,
  - *y* be ground truth,
  - $Loss_{ce}$ be Cross Entropy Loss,
  - $Loss_{dice}$ be Dice Loss,
  - $W_{ce}$ be weight assigned to Cross Entropy Loss,
  - $W_{dice}$ be weight assigned to Dice Loss,
  - $W_{d}$ be distance (to the two closest instances) weights,
  - $W_{s}$ be size weights.
  Then $W=W_{d}\cdot W_{s}$ is pixel weight and loss function has following definition:
  $$Loss\left(x,y\right)=W\cdot W_{ce}\cdot Loss_{ce}+W_{dice}\cdot Loss_{dice}$$ (1)
  It is the sum of two losses, Cross Entropy Loss and Dice Loss, each weighted by real number picked form ${\mathbb{R}}^{\left[0,1\right]}$. The Cross Entropy Loss component is additionally weighted with the pixel weight (computed as a dot product of both the distance weight and the size weight) to penalize mis-classifications on pixels belonging to the small objects and closely located instances. This lets us jointly optimize the models ability to distinguish between two closely located buildings, and also the model’s ability to segment out smaller instances. Figure 3 shows a visualization of the individual pixel weight components used in the custom loss function. These custom adaptations to the Loss Functions significantly improved the performance of our model.
3. Training Scheme: The following multi-stage training scheme along with pre-trained models (as available in PyTorch^b^) as starting points is used for better results (for both *Average Precision* and *Average Recall*):
  1. Initialize the model with pre-trained weights,
  2. Train on a 50,000 tile subset from the training set with learning rate = $10^{-4}$ and dice weight = 0.5,
  3. Train on the full dataset with learning rate = $10^{-4}$ and dice weight = 0.5,
  4. Train with a (reduced) learning rate = $10^{-5}$ and dice weight = 0.5,
  5. Train with 10 fold increase in the dice weight (5.0) to make the final predictions smoother.
4. Pre Processing
  - For each pixel: compute distances to the two closest instances are calculated to create the distance map that is used for weighing the loss function.
  - Size mask for each image is produced, that encoding the information about object size.
  - small masks on the edges of the image were dropped.
5. Post Processing:
  - Test time augmentation: Made predictions on image rotations (90–180–270°) and flips (up-down, left-right) and use the geometric mean of the predictions as the final result.
  - Second level model*.* We finally used Gradient Boosting to train a separate model using *Light-GBM* on the first stage output for computing the final prediction masks.

**FIGURE 4:**
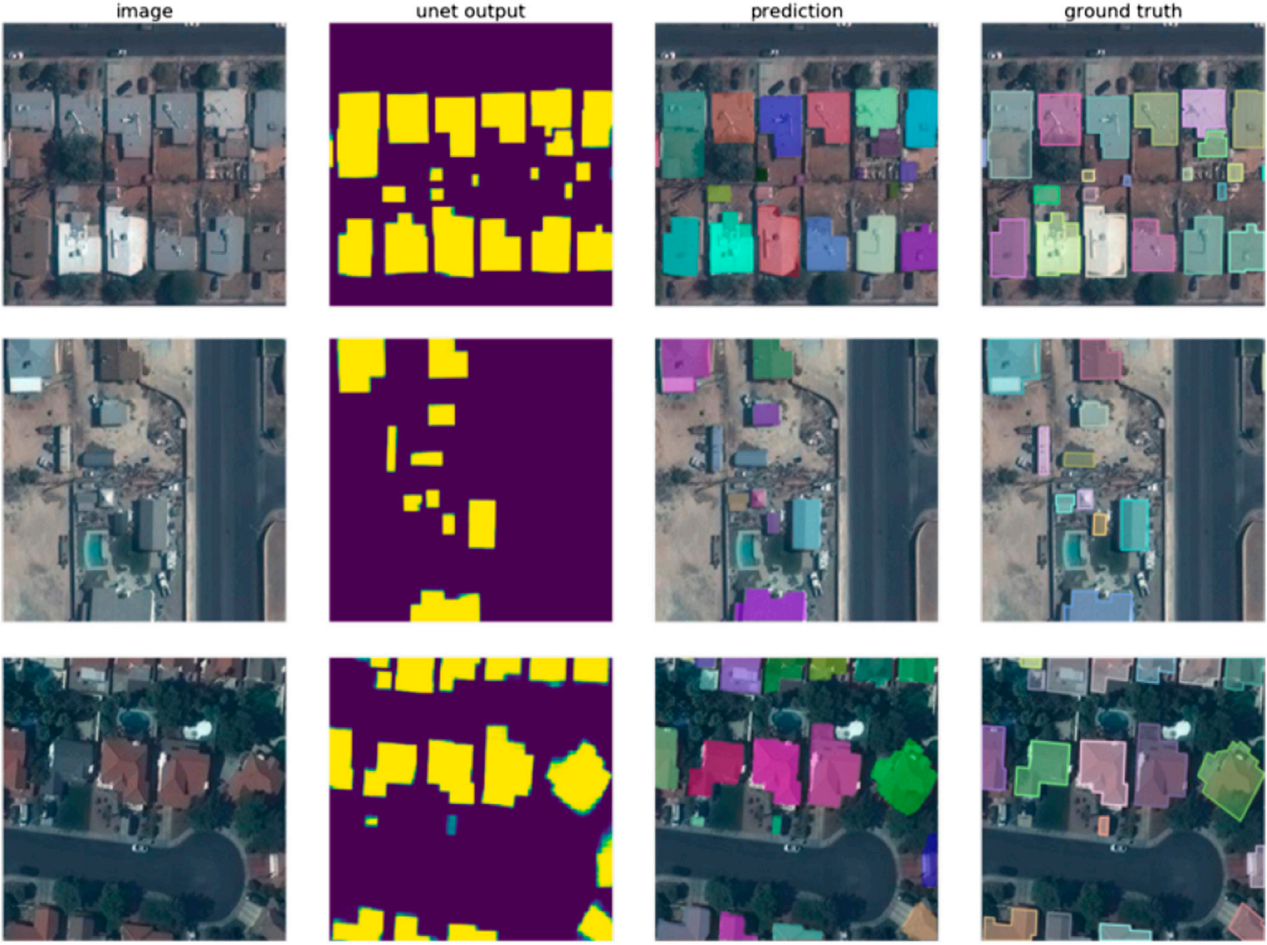
Each row represents a randomly sampled tile from the validation set; the 1^*st*^ column shows the input RGB image; the 2^*nd*^ column shows the model’^™^s prediction; and the 3^*rd*^ column is constructed as the model’^™^s prediction superimposed on the input image; finally the 4*^th^* column shows the ground truth superimposed on the input image.

Our final performance on the held-out test set was an $AP_{IoU\geq 0.5}$ of 0.938, and a $AR_{IoU\geq 0.5}$ of 0.946. Figure 4, shows examples of some predictions made by the trained model.

## 6. Fast Learnings for Fast Mapping Using U-Net

In this section, we approach the problem by reframing it as a binary semantic segmentation task. To summarize, we preprocessed ground truth labels into binary masks, trained with a 1-cycle learning rate schedule on a U-Net (Ronneberger et al., 2015) inspired architecture with an ImageNet-pretrained ResNeXt50 (Xie et al., 2016) encoder, and post-processed the probability maps into polygonized building footprint instances.

We recognized upfront that the main evaluation metric being average precision at 0.5 IoU meant that “good enough” predictions of masks that overlap each true building by greater than 50% was more important than obtaining the highest possible pixel-wise accuracy. We also kept in mind that buildings are at different scales and the smallest or portions of buildings like those cut off at the borders of an image chip would be most challenging to perform well on. These considerations factored into our decision to use a U-Net architecture with a custom loss function that works well at multiple scales and balances pixel-wise with global IoU cost optimization.

In addition to the overall objectives, we also had the goal of refining our model development process to work best with fast, lightweight models and rapid experimentation on small datasets. These criteria are equally important to on-the-field planet-monitoring work where models need to be versatile in production and robust to highly diverse datasets and use cases.

We learned quickly that experiments on the full dataset would take 1 day per experiment due to the large size of the training (260k images) and test set (60k images). Therefore, our experimentation process emphasized doing many rapid and comparable iterations using downsized and small samples of the full dataset to minimize training time per experiment. We used a smaller sample set of the data (6k train, 1.5k val, 1.8k test) and confirmed that training and local evaluation on this sample set correlated well with performance on the full train and test sets. Using this smaller sample reduced training time from 2–3 h/epoch to 3–5 min/epoch. While the 40× speed-up helped significantly, a single experiment could still take 1.5 h or more (training to 30 epochs).

Further reducing time per experiment at the expense of lower accuracy, we downsized images to 64×64, 128×128, and 256×256 and benchmarked performance at each size (i.e., number of epochs to reach a certain loss/metric, best score at end of training, epochs to converge). With downsized, smaller samples and their corresponding benchmarks, we could test new ideas as quickly as 7 min per experiment (64×64 images trained for 30 epochs) and consistently compare new experimental results against each other. The most promising experimental settings were then benchmarked on the full training data and evaluated against the held out test set. We also visually inspected predictions against ground truth regularly and made qualitative notes about common failure cases.

For experimentation, we used one remote GPU instance (Nvidia Quadro P6000 with 24 GB of GPU memory), PyTorch 0.3 with the Fast.ai library, and Anaconda Jupyter notebooks to run experiments and document results.

### 6.1. Pre Processing

Pre Processing training data consisted of reflect-padding images to 320×320 which helped increase the visible area of buildings cut off at the sides and corners of each tile. Polygon ground truth labels were converted to binary pixel masks without any other modifications. The data was augmented with random vertical/horizontal flips, 90 ± 4° rotations, and slight image brightness and contrast changes.

**FIGURE 5:**
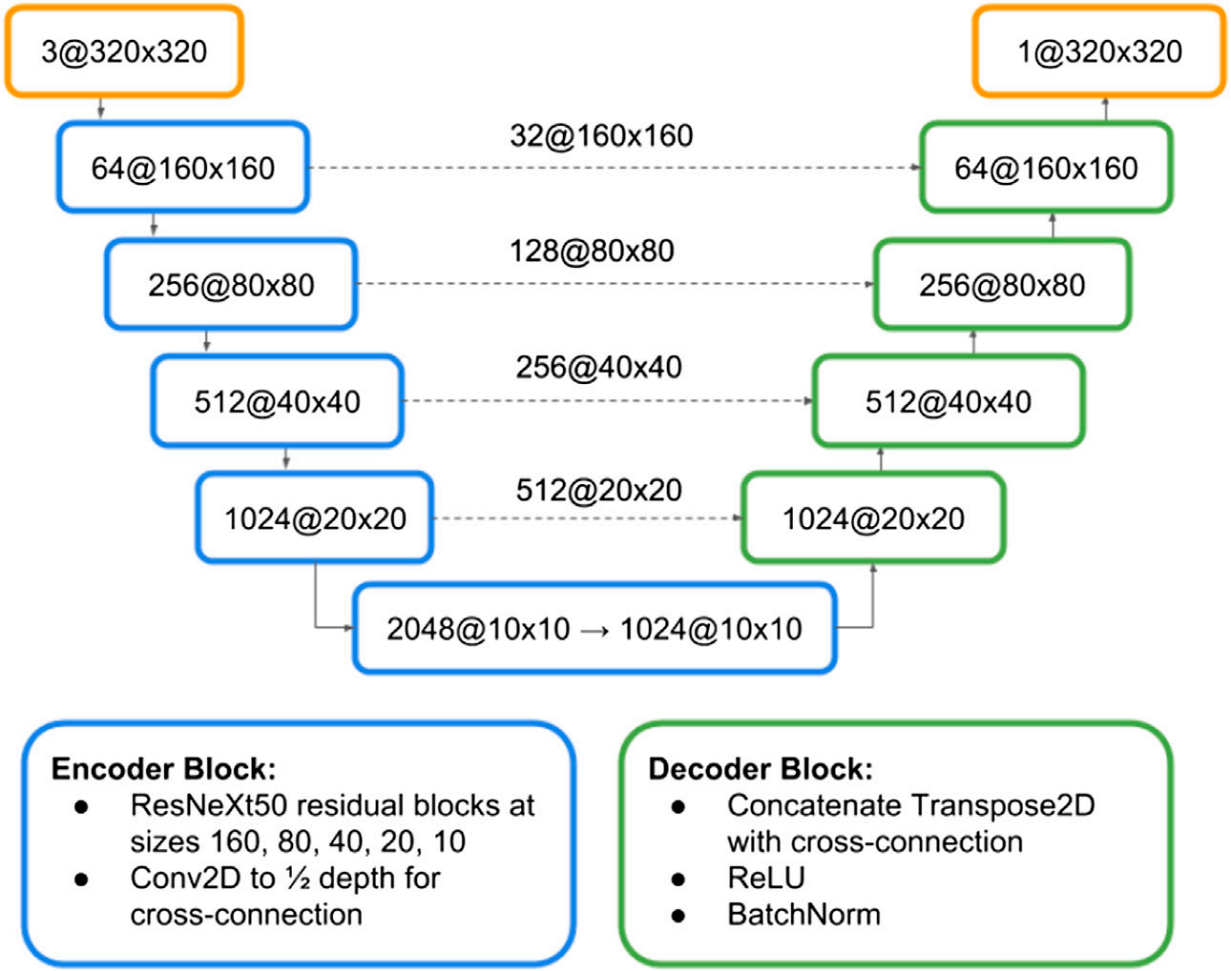
Architecture sketch for the U-Net inspired model using a ResNeXt50 encoder as described in Section 6.

Seen in Figure 5, the model architecture used is U-Net inspired with an ImageNet-pretrained ResNeXt50 encoder (weights from the PyTorch/Fast.ai library). Key characteristics include extracting features at the end of each pretrained ResNeXt block, convolutions within the cross-connections at each feature map size (160, 80, 40, 20, 10) which halves the number of channels, and upsampling using Transpose2D (deconvolution).

### 6.2. Training

The loss function is an equally weighted combination of binary cross-entropy loss and soft dice loss which empirically produced better results than either loss alone.

We trained the model with 1-cycle learning rate and cyclical learning rate schedules which was first introduced in Leslie Smith’s research on faster neural network training with very high learning rates (Leslie and Topin, 2017). In recent practical experience, 1-cycle learning was successfully employed by the Fast.ai team in Stanford’™s DAWNBench competition (Stanford DAWNBench, 2018) to achieve the fastest and cheapest methods in training CIFAR-10 and ImageNet classification models to performance benchmarks (Howard, 2018). 1-cycle training functionality is implemented directly in Fast.ai library and its experimental usage is well documented by the team (Gugger, 2017b).

**FIGURE 6:**
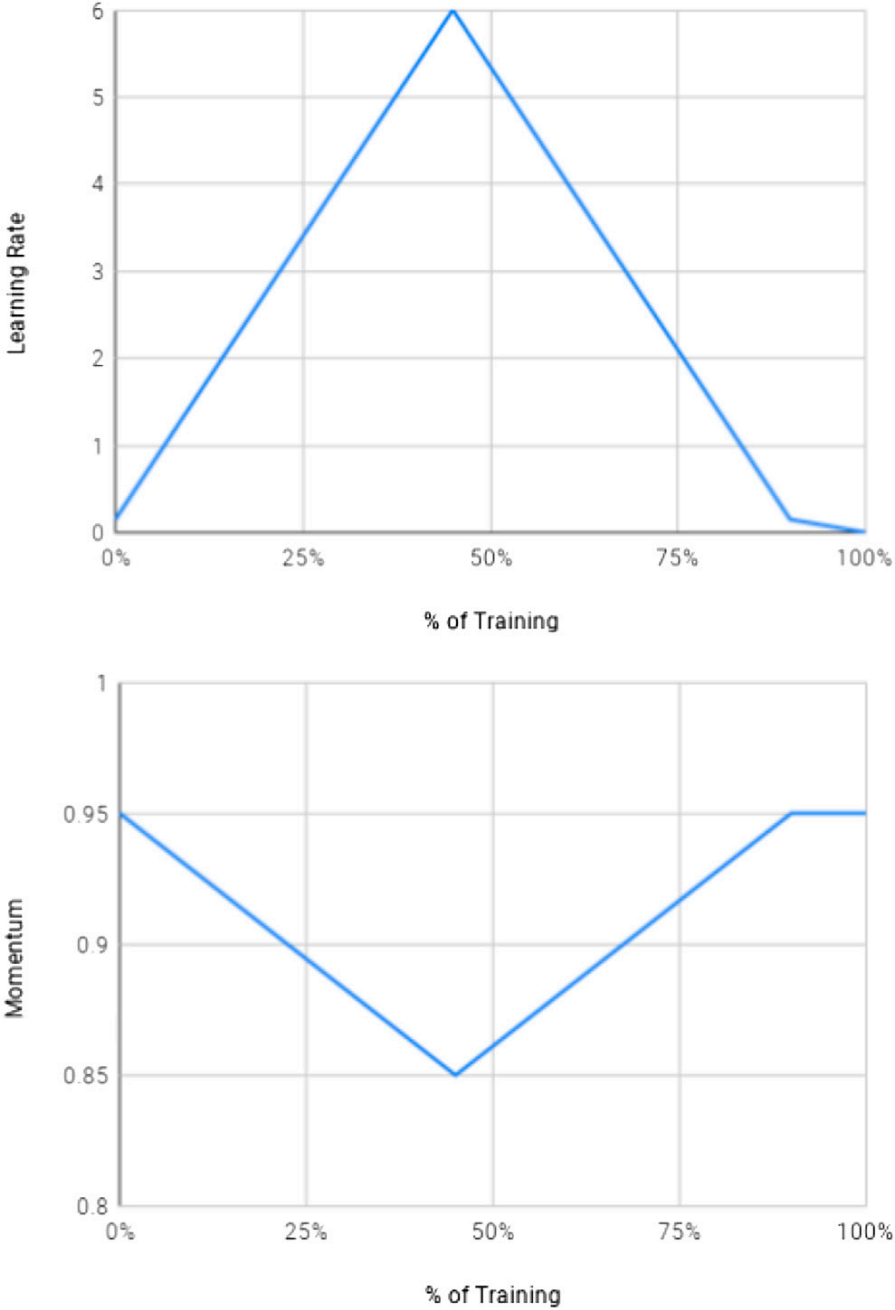
The 1-cycle schedule for learning rate and momentum over training time (as described in Section 6).

**FIGURE 7:**
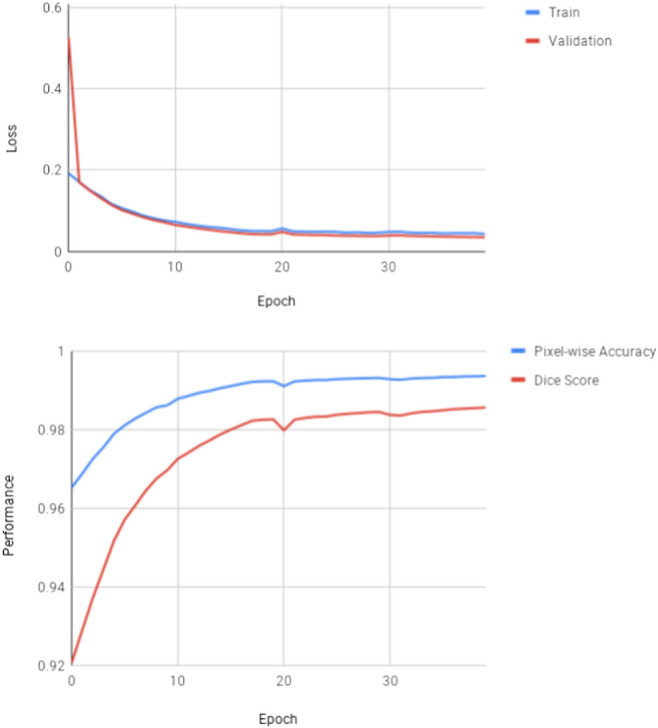
Learning curves from experiments for the U-Net adaptation using Fast Learnings for Fast Mapping (as described in Section 6).

**FIGURE 8:**
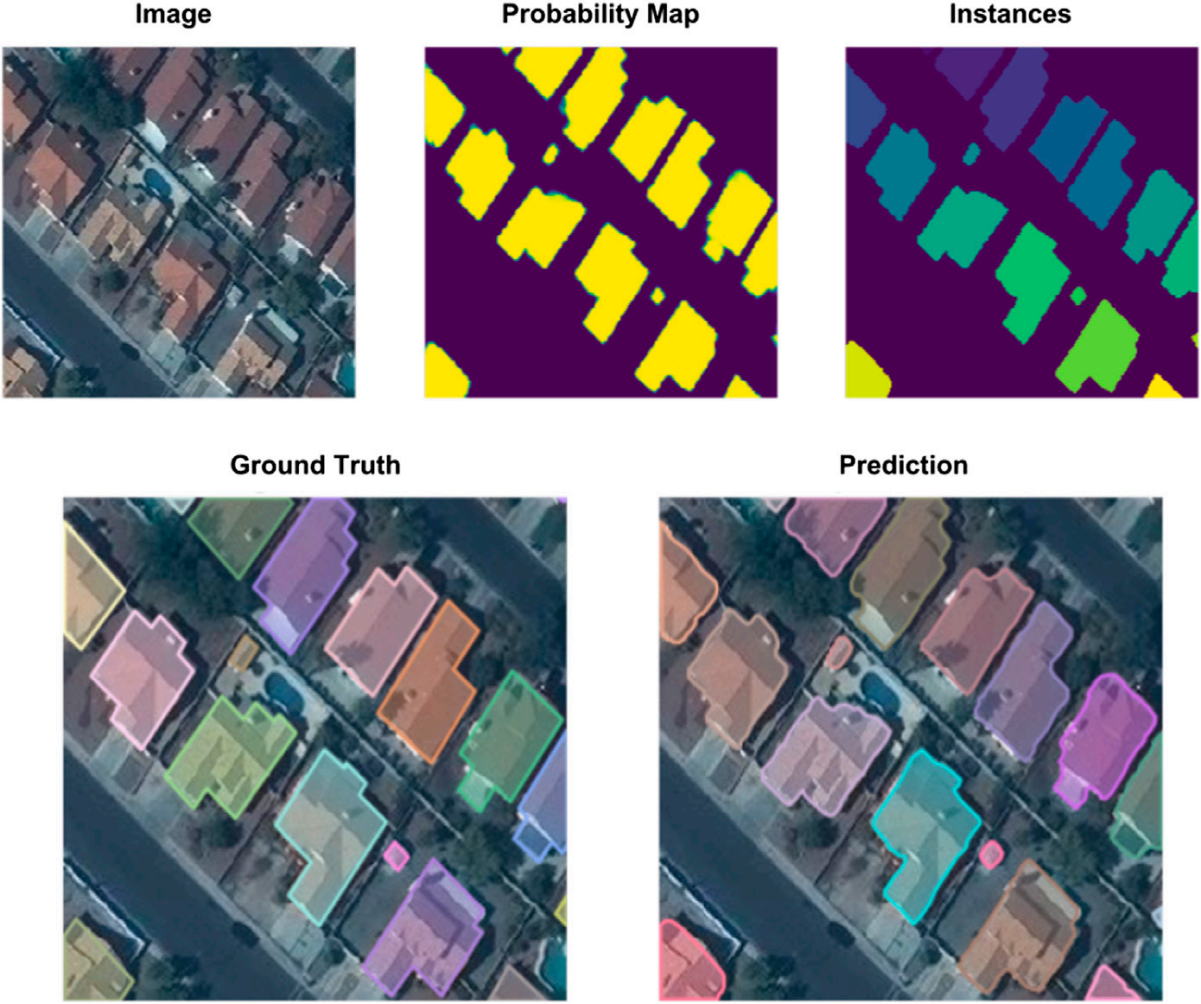
Example outputs from probability map to polygonized prediction compared with the ground truth (Fast Learnings for Fast Mapping, Section 6).

With a batch size of 32 and an optimizer of Stochastic Gradient Descent with momentum, we first warmed up the model by training the un-pretrained decoder layers for 1 epoch at a learning rate of 6. Then we unfreezed all weights and started 1-cycle training for 20 epochs as seen in Figure 6 with a learning rate of 0.15, linearly increased it to 6 by 45% through training, linearly decreased it back to 0.15 by 90% through training, and decayed the learning rate to 0.0015 in the last 10% of training. Momentum was scaled inversely to learning rate changes over the same schedule. Figures 7 and 8

After 20 epochs with this 1-cycle learning schedule, we trained for two more 10-epoch cycles with a cyclical learning rate schedule (lr = 0.05 to 1 back to 0.05 over each cycle).

Other key model training techniques employed (available out-of-box through Fast.ai library) include gradient clipping to minimize risk of gradient explosion, a learning rate finder utility to select the highest possible learning rate without divergence up front (Smith, 2017; Gugger, 2017a) and discriminative fine-tuning (Howard and Ruder, 2018) to train earlier layers at smaller learning rates than later layers.

### 6.3. Post Processing

For inference, we performed 8× test-time augmentation (every possible flip and 90° rotation) and took the geometric mean of all eight outputs to create the probability map. We used a probability threshold to create binary masks, labeled each separated mask as building footprint instances, and converted to polygon submission format. We filtered out very small areas (less than 15 pixel^2^) during polygonizing to reduce false positives. We calculated the confidence score of each building instance as the average pixel-wise probability value over the area of each instance.

As reported in Table 2, our final performance on the held-out test set was an $AP_{IoU\geq 0.5}$ of 0.918, and a $AR_{IoU\geq 0.5}$ of 0.929, with a model trained for 40 epochs total: 1-cycle for 20 epochs, 2 × 10 epochs with cyclical learning rate. After just 1-cycle training for 20 epochs, our average precision/recall was already close to best at 0.917 and 0.922. It is worth considering if the extra 20 epochs is worth the performance gain or if a single 1-cycle schedule should be used for 40 epochs instead to achieve even better results.

**TABLE 2:** An overview of experimental results: Shown is performance of the different architectures when using different loss functions during training (for the adapted U-Net architecture, Section 8).

Net. Config.	Loss	Avg. Precision (IoU)	Avg. Recall (IoU)
Net6	Binary	0.814	0.891
Net6	Binary + dice	0.828	0.899
Net8	Binary	0.878	0.919
Net8	Binary + dice	0.889	0.925
Net10	Binary	0.899	0.932
Net10	Binary + dice	0.888	0.924
Net12	Binary	0.911	0.941
Net12	Binary + dice	0.912	0.942

Toward our external objective to develop fast, lightweight models that achieve top-5 performance, our model training time was 1.6 days (1.9 h/epoch × 20 epochs) and an additional 1.6 days for the extra 20 epochs that may not have been necessary. Inference took 0.5 h per test time augmentation run to go through full held out test set.

## 7. Instance Segmentation Using Deeper U-Nets

0U-Net, as also referenced in Section 5, is an encoder-decoder network for semantic segmentation, which has its origins in medical image segmentation. The model generates a mask for the whole image. This mask then needs to be split into individual sub-masks for the separate buildings.

**FIGURE 9:**
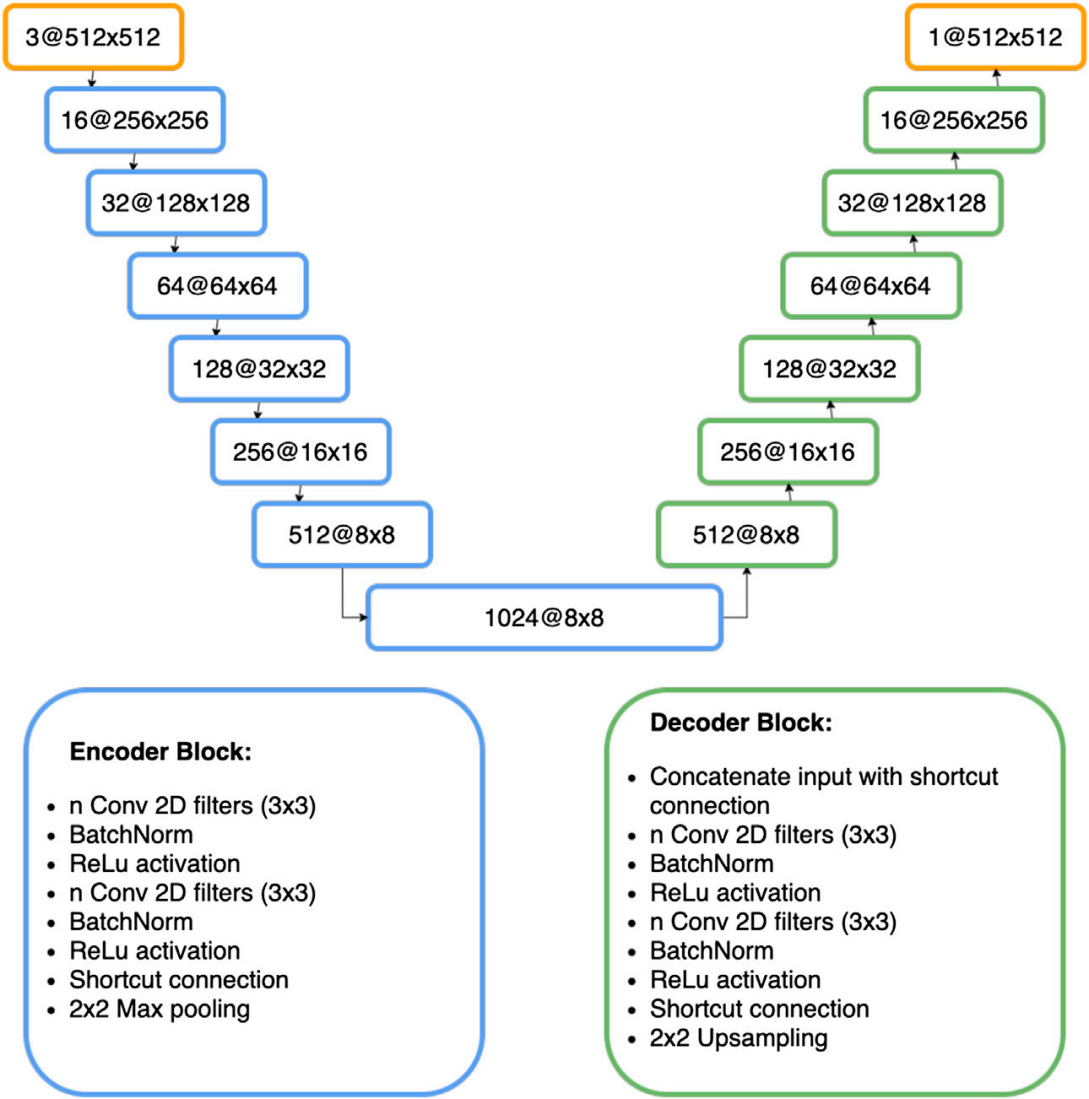
Architecture of the Deeper U-Net model (described in Section 7).

The model architecture used in this approach, is shown in Figure 9. The left half of the network (encoder) is similar to a CNN, tasked with coming up with a low dimensional dense representation of the input, and the right side (decoder) then up-samples the learned feature representations to the same shape as the input. The shortcut connections let information flow from the encoder to the decoder and help the network keeping spatial information. As the work of Li et al. (2017) has impressively shown, U-Nets benefit greatly from a deeper model architecture. It allows the model to make much more detailed segmentations. Particularly near the object borders the predictions of a deeper U-Net tend to be more accurate. We used a deep U-Net architecture which was first proposed by Giannakopoulos (2017).

The inputs are zero padded to a size of 512 × 512 in order to have an image size where 2 × 2 Max Pooling can be performed multiple times without having to deal with odd image sizes. Symmetric padding would have been another option instead of zero padding. The training images are crops of a larger satellite image. As mentioned in the previous sections, the smallest, and the most difficult objects to predict are the ones which are overflowing objects from an adjacent tile. A symmetric padding would increase the area of those buildings.

For the loss function, dice-loss is combined with binary cross entropy.

$$\begin{array}{c} ℒ=BinaryCrossEntropy+DiceLoss\\ =-\overset{n}{\underset{i=1}{\sum }}y_{true_{i}}\cdot \log\left(y_{pred_{i}}\right)+1-\frac{2|y_{pred}\cap y_{true}|}{\left|y_{pred}|+|y_{true}\right|} \end{array}$$ (2)

**FIGURE 10:**
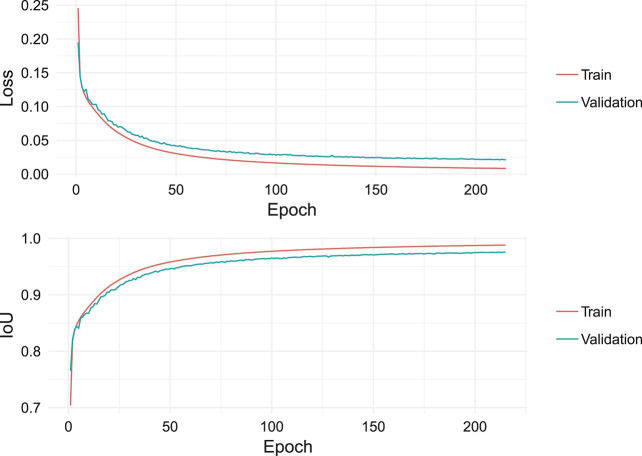
Learning curves showing loss and IoU over training time for the Deeper U-Net model (Section 7).

The model was trained from scratch for 215 epochs using RMSprop with a learning rate of 0.0001. The training took roughly 5.5 h per epoch on a single Nvidia TITAN Xp GPU. Because of the long training duration, no image augmentation was used. Interestingly the model did not start to overfit, even though no dropout is used. This is likely due to the large training size. The learning curves are displayed in Figure 10. Notice that the IoU shown in this figure, is calculated on the pixel level and therefore, not the same as IoU in the overall evaluation metric.

The model produces a probability estimate for every pixel for being an object of interest (building). A threshold of 0.5 was used, and pixel probabilities greater than 0.5 would classify a pixel as a building. The produced masks were already well separated and were easily transformed to single building masks by giving groups of pixels connected with other groups of pixels a different label. After this step masks with less than 25 pixels were deleted. This removes little artifacts that should not be counted as buildings. A good drop off pixel threshold was empirically computed by looking at the distribution of the areas of the small objects in the dataset. Then we calculate the bounding boxes for every building mask. The building masks and the bounding boxes finally form the final predictions.

Our final performance on the held-out test set was an $AP_{IoU\geq 0.5}$ of 0.930, and a $AR_{IoU\geq 0.5}$ of 0.956.

**FIGURE 11:**
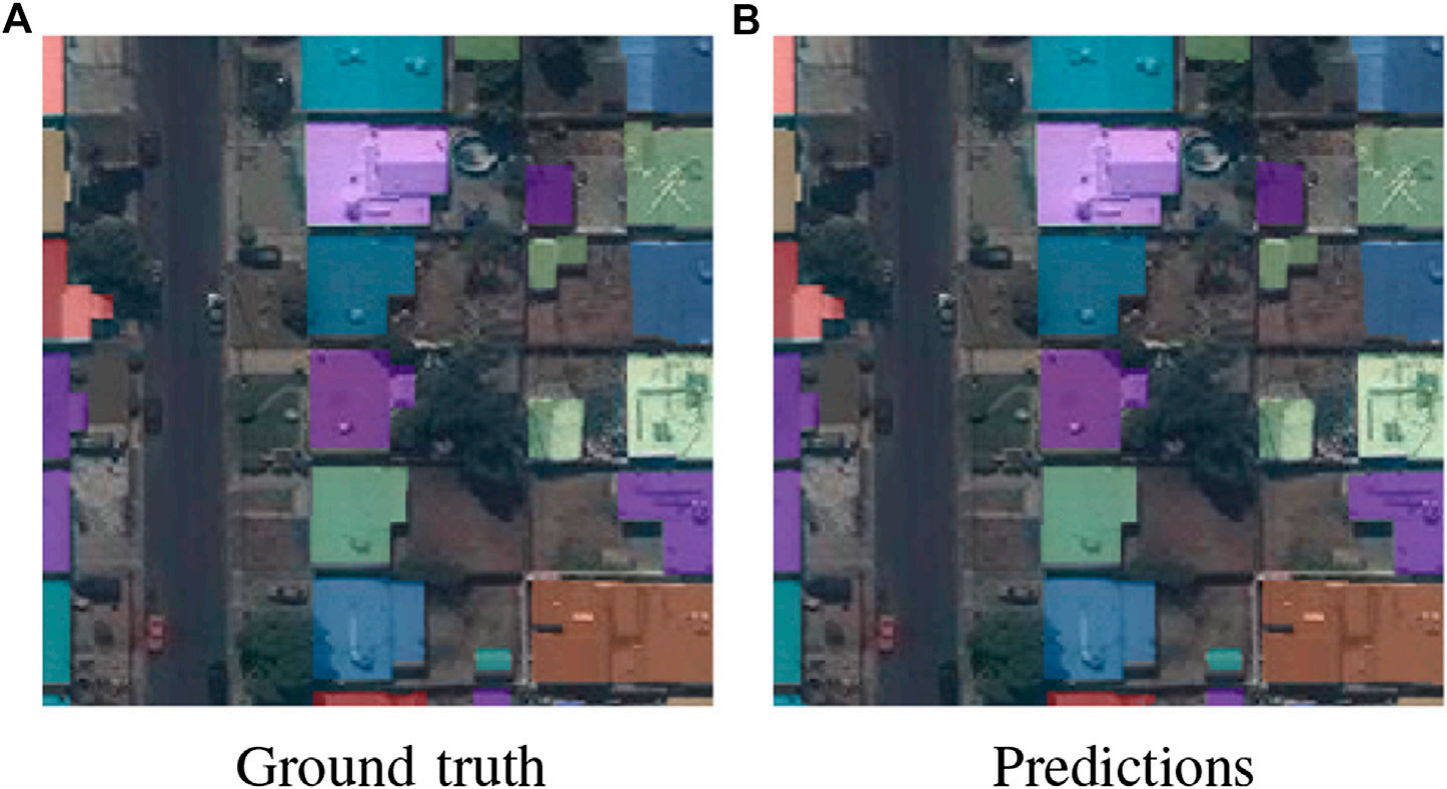
Comparing constructed masks, drawn by a human **(A)** and predicted by the Deeper U-Net model **(B)**.

Figure 11 shows a prediction using the Deeper U-Nets, for a sample image from the validation set.

The generated masks are very accurate and it is expected, that the model would also perform well on other categories such as roads, trees, crops, rivers and lakes. This could make todays Mapathons, where volunteers draw maps from satellite images, completely obsolete. Instead of days or even weeks, maps could be generated in just a few hours from satellite imagery or drone footage. This is crucial for emergency preparedness actors who go to remote areas where no maps exist. Up-to-date maps help them to work efficiently in a crisis situation such as an earthquake.

As the work of Li et al. (2017) has impressively shown, U-Nets benefit greatly from a deeper model architecture. It allows the model to make much more detailed segmentations. Particularly near the object borders the predictions of a deeper U-Net tend to be more accurate.

## 8. Comparing an Adapted U-Net Architecture for Varying Depths

In this section, we are analyzing and comparing a U-Net like structure (Ronneberger et al., 2015) for different depths. The architecture was derived originally from a convolutional AutoEncoder structure as used for reconstructing images (see, for an example the keras tutorial Chollet (2015)). This AutoEncoder-type architecture was modified for semantic segmentation: the provided ground truth annotations of the buildings were used as targets for training in order to accomplish the detection of buildings. Furthermore, skip-connections were introduced as found in U-Net that connect encoding and decoding blocks on the same level. These connections help to recover spatial information and in our experiments this provided better reconstruction of details in images compared to post-processing, for example, using conditional random fields (Krähenbühl and Koltun, 2012). This architecture differs from U-Net, first, with respect to the sequence inside the decoding blocks. Following the AutoEncoder approach, the decoder block mimics exactly the encoder block and consists of a single convolution followed by upsampling (max-pooling is used in the encoder block). Second, we used a single convolution of size 5×5. This architecture was used for different depth (stacked blocks of encoders and decoders).

In the first part, we speak about the applied pre- and post-processing of the data-set. Secondly, we introduce our designed network architecture in Section 8.3 and explain our training procedure. In Section 8.5, results are presented that compare variations of our architecture. The results are then summarized and discussed in Section 10.

### 8.1. Pre-processing

We normalize each input image individually by subtracting its mean and dividing by its standard deviation.

For early tests we resized the images down from 300 × 300 pixels to 128 × 128 pixels, in order to avoid long training times. For our final models, we ultimately used the full resolution so as not to lose any detail.

### 8.2. Post-processing

For each of the 300 × 300 pixels in an input image, the networks yields a pseudo-probability between 0 and 1, where low values correspond to background (i.e., no building) and high values correspond to foreground (i.e., building).

We binarized these values by setting a threshold *θ* and assigning 1 if the value is bigger than the threshold *θ* and 0 otherwise.

To address noise in the background we perform a morphological opening (as provided by OpenCV Bradski, 2000) with a radius of 1, that is an erosion (which chooses the minimum value of a neighborhood) followed by a dilation (which chooses the maximum value). This also helps separate closely connected buildings.

In the initial experiments, we tried to apply CRF for post-processing (Krähenbühl and Koltun, 2012), as those have been successfully used for semantic segmentation before. In CRF, the output values are made dependent directly on characteristics in the input image, such as edges. Efficient inference on fully-connected CRF models leads to much finer structure in the output. While in many applications this allows recognition of small details, it did not help us in our use case of detecting buildings in aerial images. In fact, the results were worse when applying CRF. After looking closely at the dataset, we noticed that the desired output maps do not contain particularly fine structures, but are instead fairly regular and in most cases contain rectangular buildings. Aerial images, by contrast, contain fine details (e.g., created by shadows), which a CRF-based post-processing tries to integrate. This seemed to damage the overall performance in our case. Instead, for post-processing, it proved to be more important to establish the general structure of buildings, which meant filling in small holes inside of larger areas that have been recognized as a building and which might appear as a result, for example, from occlusion.

### 8.3. Network Architecture

**FIGURE 12:**
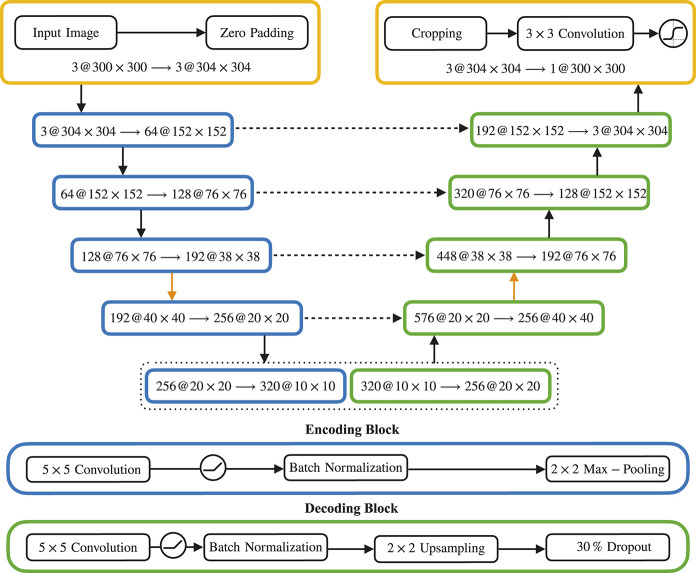
**Figure 15**: Schematic representation of our U-Net-based network architecture (Net10): A sequence of encoding blocks (in blue) on the left and a corresponding sequence of decoding blocks (in green) on the right, with skip connections (dashed arrows) between them. Inside each block, we indicate the size of the internal representation at that stage. We show the setup of all encoding and decoding blocks at the bottom. (Comparing an adapted U-Net architecture for Varying Depths).

Our network consists of a series of encoding blocks, followed by as many decoding blocks (see Figure 12). We tried networks with 6, 8, 10, and 12, which we refer to as Net6, Net8, Net10, and Net12, respectively. The deeper the network, i.e. the more blocks it contains, the better it performs. For this particular work, our best evaluated results were from Net12.

The composition of individual blocks always follows the same structure:

- Encoding blocks consist of a 5×5 convolution layer (padded with a stride of 1×1) with an increasing number of 64, 128, 192, 256, and 320 filters, followed by a Rectified Linear Unit. The resulting features are then normalized using batch normalization (Ioffe and Szegedy, 2015), and downsampled by a 2×2 max pooling operation.
- Decoding blocks are symmetric to the encoding blocks, also using a 5×5 convolution (padded with a stride of 1×1), followed by a Rectified Linear Unit, batch normalization and a 2×2 upsampling operation, where the low dimensional features of the previous layer are resized. Additionally, a dropout layer (Srivastava et al., 2014), omitting 30% of the neurons, is added in the decoding blocks.

Additionally, we integrate skip connections (Ronneberger et al., 2015). For this, the outputs of corresponding (see Figure 12) encoding and decoding blocks are concatenated and fed as inputs to the next block. The introduction of skip connections improved results greatly, even during our initial experiments with the downsampled data. In particular, skip connections helped bringing out more detailed structures of buildings.

Our approach is similar to Segnet (Badrinarayanan et al., 2015) which is also using block-wise encoders and decoders, as well as upsampling on the decoder side. In contrast to our approach, they are always applying multiple convolutions, but of a smaller size. In addition, during decoding, the convolutions are applied before the upsampling step. Furthermore, in SegNet, the indices of the max-pooling layer are used during upsampling, while in our approach, information flows using the skip connections, much like U-Net.

As the overall goal was the detection of buildings within the input images, we set up the network as a binary classifier. Therefore, after the last convolution, we use a sigmoid activation function, to obtain pixel-wise pseudo-probabilities, as discussed above.

In order to match input and output size after downsampling and upsampling, we use zero padding and cropping padding when necessary.

### 8.4. Training

For the implementation and training of our approach, we used keras (Chollet, 2015) while using tensorflow (Abadi et al., 2016) as the backend framework for training our models on multiple GPUs. The same procedure was followed to train different network configurations Net6, Net8, Net10, and Net12 that differ in depth. The task for all the variations of the architecture was to map the 3-channel training data to the corresponding 1-channel ground truth annotation. We adopted the ADAM (Kingma and Ba, 2014) optimizer with the default settings: beta_1 of value 0.9 and beta_2 of value 0.999 using mini-batch gradient descent. For the network configuration Net6, Net8, and Net10, the initial learning rate was set to $\alpha =10^{-3}$. At every training-step *t*, the learning rate was decayed according to $\alpha \leftarrow \alpha \cdot {\left(1+\delta_{\alpha }\cdot t\right)}^{-1}$ with a decay rate of $\delta_{\alpha }=5\times 10^{-5}$. For Net12 the learning rate was also set to an initial value of $\alpha =10^{-3}$, but no decay was applied. The batch size was varied as 32 or 64 based on the network configuration with the maximum utilization of 2 × NVIDIA Tesla p100 or 2 × GeForce GTX 1080 Ti GPUs respectively. The replicated network’s weights were merged on the local CPU on end of each epoch.

For the first 50 epochs all networks were trained using the *binary cross-entropy*. For further refinement, the networks were trained for 10 additional epochs with the *dice coefficient* loss (Milletari et al., 2016).

Using the setup that was described in this section, the average training time for each network Net6, Net8, Net10, and Net12 took about 2.2 days.

As we considered this challenge as a binary classification problem (buildings and non-building classes), the binary cross-entropy will be defined as

$$ℒ=-y_{t}\text{log}y_{p}-\left(1-y_{t}\right)\text{log}\left(1-y_{p}\right),$$

where $y_{t}$ is the target and $y_{p}$ the predicted class.

### 8.5. Experiments and Results

The four in this section presented network architectures of varying depths were trained on the provided dataset according to the training procedure described in Section 8.4. To find the optimal parameters, the threshold (*θ*) value was varied from 0.2 to 0.95 with respective squared shape morphology kernel size (*k*) between 0 and 5. Even though the pixel-wise accuracy was high because of the dominating background class, the mis-classified pixels lead to decrease in precision and recall.

An overview of the final results are reported in Table 3 with morphology for dilation and erosion set to 1. In the table, evaluation results are shown as scored on the official test data set for all networks of varying depths.

**TABLE 3:** Precision and recall per epoch at detection (NMS THRESHOLD = 0.5 for the full validation set).

Epoch	Average precision	Average recall
1	0.8989	0.9240
2	0.9020	0.9274
3	0.9137	0.9381
4	0.9233	0.9443
5	0.9350	0.9514
6	0.9359	0.9528
7	0.9367	0.9546
8	0.9370	0.9554
8 (TTA)	0.9374	0.9574
(0.4 Thres)		
8 (TTA)	0.9438	0.961
(0.5 Thres)		

The network configurations Net6, Net8, Net10, and Net12 are evaluated with *θ* = 0.5. The listed results in Table 3 show that the precision of the network was improved when the number of used encoding and decoding blocks is increased. This might indicate that a further improvement of the precision in the detection of buildings can be achieved by again enlarging the network architecture with more blocks.

The additional training with the *dice coefficient* did only slightly enhance the performance of the networks Net6, Net8 and Net12, but not in the case of Net10.

As reported in Table 3, our final performance on the held-out test set was an $AP_{IoU\geq 0.5}$ of 0.912, and a $AR_{IoU\geq 0.5}$ of 0.942.

## 9. Instance Segmentation using Mask R-Convolutional Neuronal Networks

In this section, we explore the use of Mask R-CNN, a two-stage object detection architecture to detect a single class (buildings). Here, we consider Mask R-CNN in contrast to U-Net based approaches, as an IoU threshold of 0.5, helps to not warrant the high semantic accuracy that U-Net based pixel classification approaches provide. On the other hand single stage object detection architectures such as SSD (Liu et al., 2016), simply learn bounding box regression and its class probabilities. They have faster inference times, but Mask R-CNN has consistently shown better accuracies, and includes semantic output.

**FIGURE 13:**
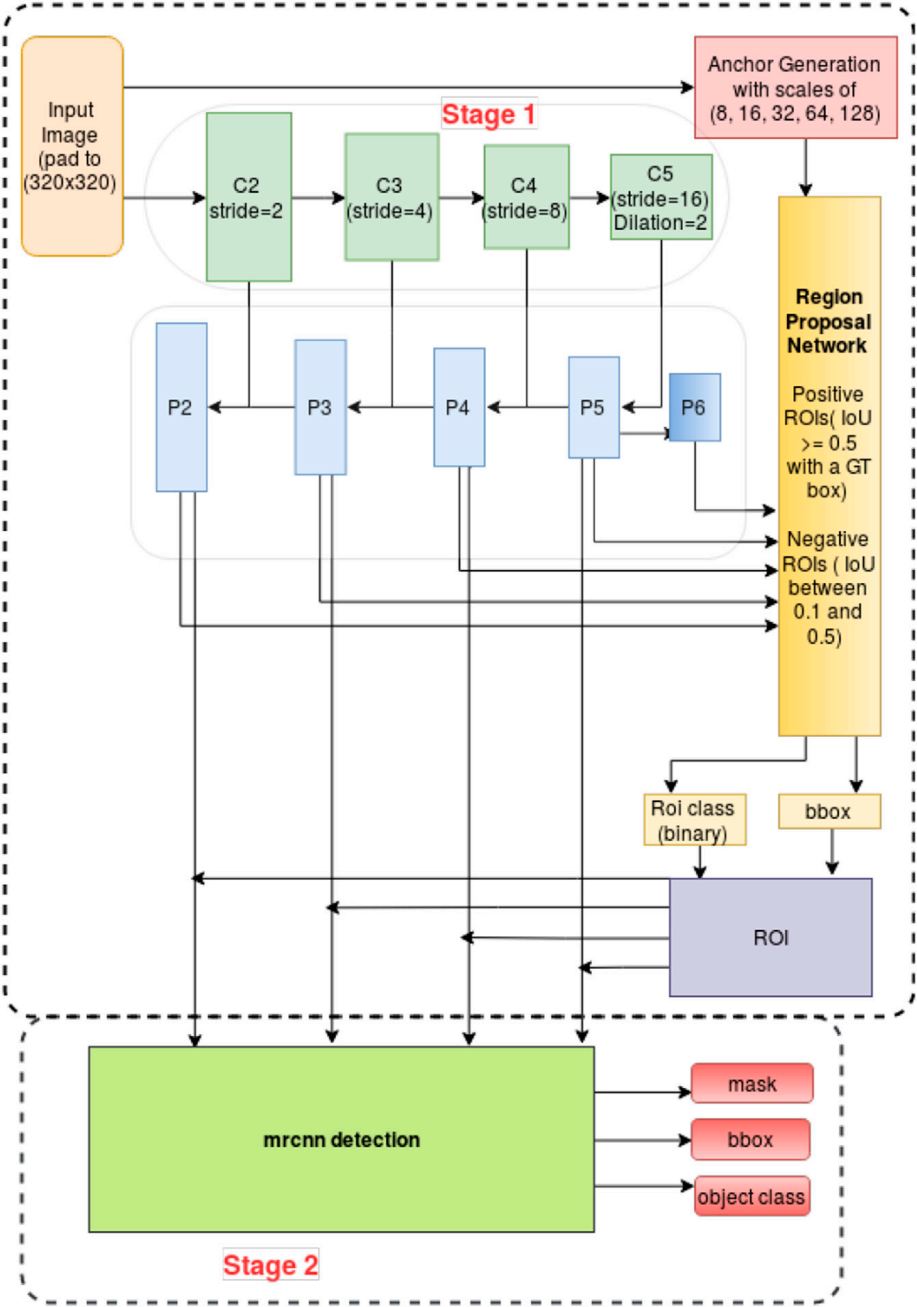
Mask R-CNN stages with modifications introduced for the Mapping challenge (see Section 9 for detailed explanation of architecture and modifications).

Mask R-CNN builds up on the Regional Proposal Networks as proposed in Faster RCNN (Ren et al., 2015). This first stage proposal network (illustrated in Figure 13) selects the regions of interest from a pre-determined set of anchors, and feature-maps from a bottom-up Resnet-101 backbone (He et al., 2016), and a top-down feature pyramid network (Lin et al., 2017). It matches Anchors of different sizes and aspect ratios, computed from a set of scales, to objects in an image. For implementing the modifications upon Mask R-CNN, we start off with open source implementation of Mask R-CNN (Matterport, Abdulla, 2017).

**FIGURE 14:**
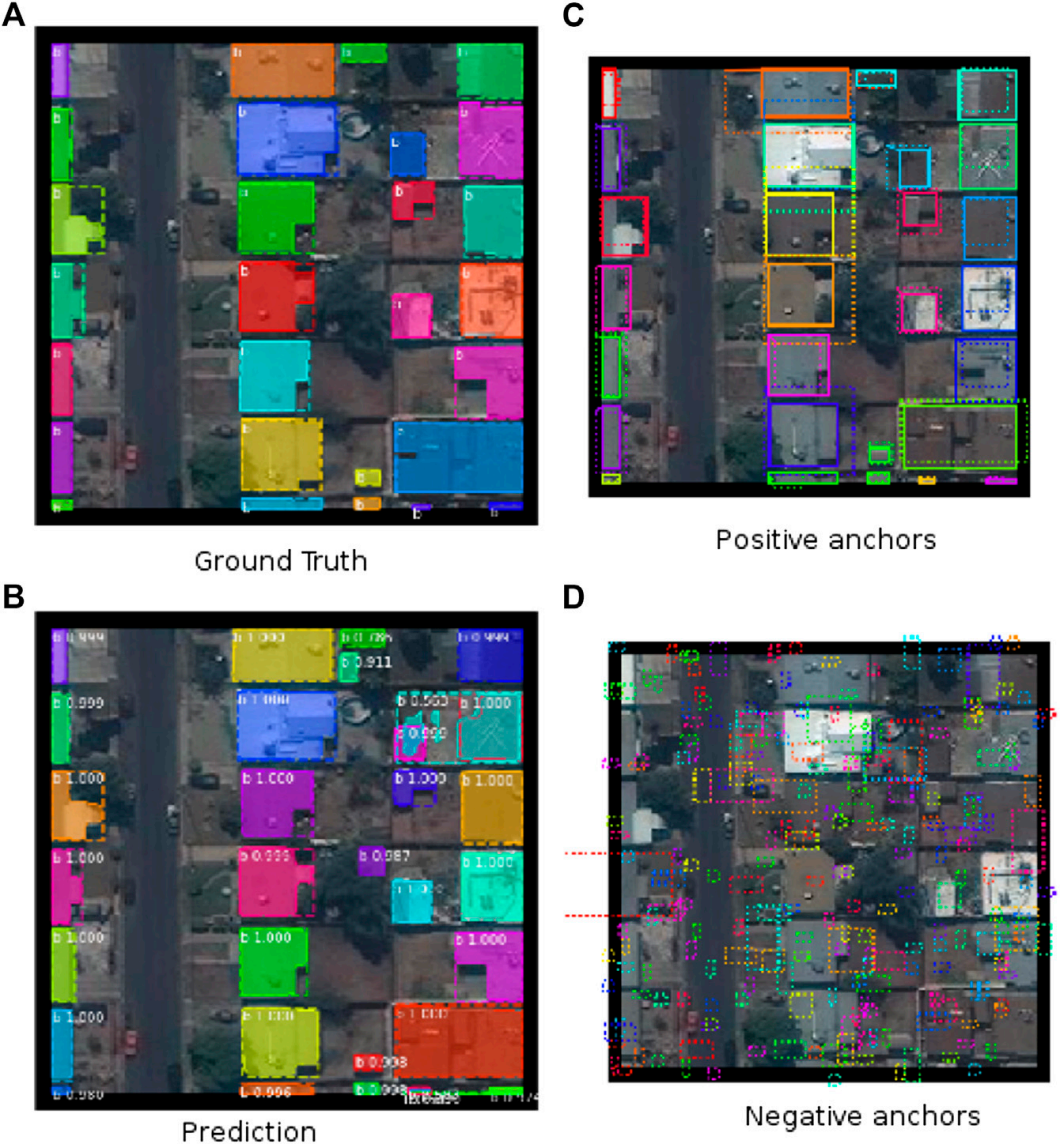
Intermediate anchor generation from region proposals and final mask predictions from the detection stage for the Mask R-CNN approach.

For the task of instance segmentation on satellite imagery, we considered anchor scales of 8, 16, 32, 64, 128. We considered smaller anchor sizes as the dataset had a significantly higher distribution of “small” instances, and at the same time the input images had a maximum size of 300×300 pixels. The distribution of instances in the training and the validation set includes 60% of the instances were medium sized instances (area between 1,024 and 9,216 pixel^2^), and 37% instances were small instances (area less than 1,024 pixel^2^). 19% of the total annotations had an area less than 256 pixel^2^ (in many cases = 16%, because of tile borders intersecting the edges of the buildings). The changed anchor size ensures that the regional proposal network appropriates anchors suitable, specifically for small objects. A montage containing the generated anchors, refined anchors with small deltas, ground truth, and prediction for an image is shown in Figure 14.

The second stage of detection in Mask R-CNN, RoIAlign, a novel feature introduced in Mask R-CNN aligns the feature-maps from first stage to the input image, resulting in better localization of output masks.

Mask R-CNN implementation creates a placeholder for the ground-truth masks that can consume a large amount of memory. Up-sampling the image, will create a placeholder for masks with the up-sampled dimension. Instead changing the stride as mentioned, means lesser memory requirements. At the same time, with the reduction in down-sampling, the overall compute overhead increases. On an Amazon AWS P3 instance (p3.2xlarge), which uses a single Nvidia V100 T GPU with 16 Gb GPU memory, an epoch with a batch size of 4, takes roughly 16 h.

In the Mask R-CNN implementation, negative anchors out of the ROIs, are assigned when IoU is less than 0.3 and positive when IoU is greater than or equal to 0.7, compared to the ground-truth boxes. To generate targets for Stage 2 classifier and mask heads, without using the RPN head, the default implementation uses 0.5 threshold to distinguish between negative ROIs and positive ROIs. Assigning negative values to ROIs with IoU of 0.1–0.5, ensures that there is some minimum intersection with the ground truth. ROIs that have less than 0.1 IoU are discarded. Doing so introduces hard example mining, as even to form negative samples, there is some minimal intersection criteria. We padded the images by 10 pixel on each side to create the final input image of 320×320 pixels. Padding ensures that at the border there are more valid anchors available to select the best matching ROIs, including the elongated instances that have a small width or height.

One potential avenue to improve small object detection is to use dilation (Yu and Koltun, 2015), which enlarges the receptive field without losing resolution, and so can provide a context for detecting small buildings. We set the dilation rate to 2 on all combination of stages in the Resnet backbone. This is a dataset for a single object detection, so discriminative information from context could not perhaps be as useful but providing the context, in general, is a valuable means in detecting small objects.

To train on the Mapping Challenge Dataset, we used the pre-trained model (trained on MS COCO dataset) to initialize the Mask R-CNN network. Then we used the Mapping Challenge Dataset to train the model for eight epochs, as shown in Table 4. Epoch 1 to 4 minimized the loss using Stochastic Gradient Descent, at a learning rate of 0.001 and Epoch 5 to 7 used learning rate of 0.0001. On epoch 8, we used half the samples to train at a learning rate of 0.0001 but the other half used learning rate of 1e−5. We also applied an augmentation of horizontal flip and a vertical flip during training.

**TABLE 4:** Overview and comparison of results for each model.

Metric	Model 1	Model 2	Model 3	Model 4	Model 5
(AP) @[ IoU = 0.50:0.95 — area = all — maxDets = 100 ]	0.839	0.720	0.799	0.679	0.665
(AP) @[ IoU = 0.50 — area = all — maxDets = 100 ]	0.930	0.900	0.938	0.889	0.937
(AP) @[ IoU = 0.75 — area = all — maxDets = 100 ]	0.886	0.780	0.865	0.752	0.817
(AP) @[ IoU = 0.50:0.95 — area = small — maxDets = 100 ]	0.662	0.441	0.560	0.398	0.501
(AP) @[ IoU = 0.50:0.95 — area = medium — maxDets = 100 ]	0.959	0.897	0.924	0.860	0.741
(AP) @[ IoU = 0.50:0.95 — area = large — maxDets = 100 ]	0.943	0.923	0.965	0.911	0.752
(AR) @[ IoU = 0.50:0.95 — area = all — maxDets = 1 ]	0.109	0.094	0.113	0.090	0.094
(AR) @[ IoU = 0.50:0.95 — area = all — maxDets = 10 ]	0.750	0.678	0.709	0.650	0.608
(AR) @[ IoU = 0.50:0.95 — area = all — maxDets = 100 ]	0.905	0.810	0.819	0.773	0.722
(AR) @[ IoU = 0.50:0.95 — area = small — maxDets = 100 ]	0.776	0.588	0.601	0.541	0.599
(AR) @[ IoU = 0.50:0.95 — area = medium — maxDets = 100 ]	0.982	0.940	0.946	0.908	0.794
AR) @[ IoU = 0.50:0.95 — area = large — maxDets = 100 ]	0.980	0.970	0.978	0.963	0.819

Model 1 - Instance segmentation using Deeper U-Net (Section 7); Model 2 - Instance Segmentation using Mask R-CNN (Section 9); Model 3 - Adapted U-Net architecture of Varying Depths (Section 8); Model 4 - Fast Learnings for Fast Mapping Using U-Net (Section 6); Model 5 - Instance Segmentation Using Customized U-Net (Section 5).

**FIGURE 15:**
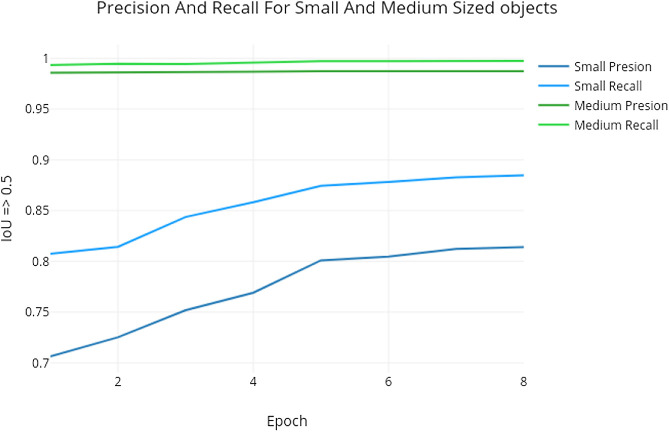
Comparison of precision and recall for different object sizes when using the Mask R-CNN approach detailed in Section 9.

Epoch 2 to 4 were trained on a smaller subset of the training set, obtained by filtering the dataset so that at least one object of area less than 256 ${\text{pixel}}^{2}$ appeared in any given image. Medium and large objects already had high precision and recall at epoch 1, as shown in Figure 15.

Nevertheless, We can see in Figure 15 that from epoch 1 to epoch 8 small object precision improved from 0.706 to 0.82 while medium object improved marginally from 0.9857 to 0.9874. An explanation can be attributed to the fact that the smallest anchor scale used for the MS COCO dataset was 32, while in ours, the smaller anchor scale of 8. While the hierarchical representation learned by the ResNet backbone on the COCO dataset, especially in the bottom layers were useful, the Mask R-CNN model had not seen many small object samples, so the whole pipeline required more training to be as effective for the smaller objects. Weights trained from samples with mask loss weight set to 10 was also kept as part of the final model. A straightforward approach that might result in a better model would be to train for four epochs with a learning rate of 0.001, another 4 with a learning rate of 0.0001 and 1 epoch with learning rate of 1e−5, with no sub-setting and with no changes to mask loss weights. At validation and test time, predictions from the annotated images, flipped vertically and horizontally is merged with the regular prediction. Then *non-max-suppression* is applied to obtain the final prediction annotation.

The multi-task loss in Mask R-CNN includes loss from the region proposal stage and the detection stage. The regional proposal loss consists of, *class loss*, for positive and negative ROIs and associated *bounding box loss*. The detection stage consists of, *object class loss*, *bounding box loss* and *mask loss*. Weighting a particular loss can affect its contribution to the overall loss. In this implementation, we increased the weight of the mask loss by 10 folds, near the end of training, for better semantic segmentation.

Our final performance on the held-out test set was an $AP_{IoU\geq 0.5}$ of 0.937, and a $AR_{IoU\geq 0.5}$ of 0.959.

## 10. Conclusion

In this work, we explore different flavors of U-Net and Mask R-CNN on a task of instance segmentation on high resolution satellite imagery to detect buildings. The dataset used, was a derivative of the SpaceNet (Spacenet on aws, 2018) dataset, and was post processed to enhance ease of accessibility for a broader set of Deep Learning researchers who may or may not be familiar with the handling and manipulation of raw satellite imagery. The evaluation metric used for all the experiments was designed to incentivize loose segmentation (an IoU≥0.5 was considered a correct detection) of buildings of various shapes and sizes.

**FIGURE 16:**
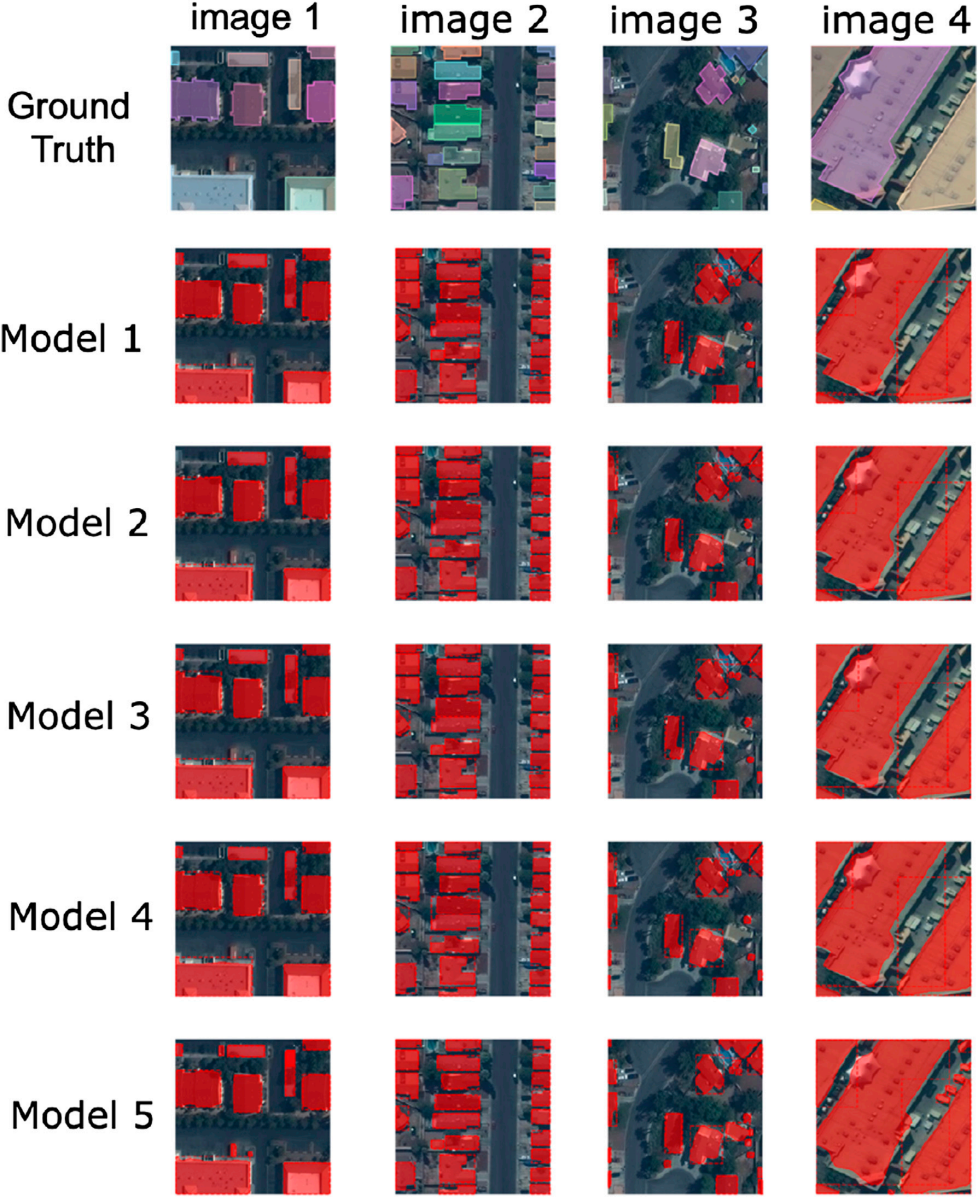
Inference results of each model on a few sample images: Model 1 - Instance segmentation using Deeper U-Net (Section 7); Model 2 - Instance Segmentation using Mask R-CNN (Section 9); Model 3 - Adapted U-Net architecture of Varying Depths (Section 8); Model 4 - Fast Learnings for Fast Mapping Using U-Net (Section 6); Model 5 - Instance Segmentation Using Customized U-Net (Section 5).

In the previous sections, four U-Net implementations were presented, each coming with its own specificities, and one Mask R-CNN approach, which was finally found to be the best performing model. A comparison of some example results for all these different architectures is shown in Figure 16 and detailed results on a test data set are given in Table 5.

**TABLE 5:** Explanation of computational steps of the U-Net pipeline (see Figure 2).

Step name	Description
Input	RGB images of 300 × 300 pixels
Specs	Meta-data that serves as second input file
xy_inference	Step that extracts all paths to the input data form the meta-data
tta_generator	Step that prepares test time augmentation (*tta*) routines
Loader	PyTorch loaders, that compute batches of data
Unet	Neural network that is being trained
tta_aggregator	Step that aggregates *tta* results
prediction_renamed	Trivial step that change input names
mask_resize	Step that resizes masks from 256×256 to (original) size 300×300
category_mapper	Step that assigns class to the mask
mask_erosion	Step that performs masks erosion
Labeler	Step that applies label to instances
mask_dilation	Step that performs masks dilation
score_builder	Step that calculates scores needed for the submission purposes
Output	Output from the pipeline with all masks

The task presented two major difficulties arising from the evaluation metric choice: the designed model had to detect small instances as effectively as larger ones, and had to distinguish thin separation areas between closely located buildings. The first U-Net approach, presented in Section 5, relied on a custom weighted loss function to alleviate these difficulties, penalizing more misclassification of pixels located on small instances or in the separation areas of two close buildings. At testing time, several images were generated by rotating and flipping the original ones, and a gradient-boosting algorithm—Light-GBM—was used to construct the final prediction mask, based on the aggregated U-Net outputs. Although this approach allowed to reach high performance scores ($AP_{IoU\geq 0.5}$ of 0.938, $AR_{IoU\geq 0.5}$ of 0.946), another U-Net approach, presented in Section 7, achieves similar scores without performing any data augmentation, introducing custom weight coefficients in the loss, nor using boosting algorithm on top of the results. The new U-Net proposed was however modified to become one layer deeper compared to the original U-Net implementation, which increased the training time needed. One approach that originated from an auto-encoder-like structure systematically analyzed the influence of the depth of a U-Net like architecture in Section 8. It further investigated additional improvements on top of the auto-encoder network as the use of some image processing tools, like the morphological opening filter and CRF. Deeper U-Nets showed to further improve the performance, but not only is training time increasing, but the impact grew smaller. This might be further enhanced through introducing data augmentation as done in the first approach and which might become more important with a growing number of layers and parameters. In Section 6, another U-Net is presented, applying other adaptations during training: it was trained following customized learning rate and momentum schedules, allowing to reduce the time needed to train the model until convergence. Both these two U-Net approaches allowed to reach high scores similar to the ones that were attained with the two approaches presented first. Finally, the best performing model is the one presented in Section 9, which proposes an implementation of Mask R-CNN to solve the segmentation task. To improve detection of small instances, the anchor sizes were lowered compared to the original implementation. This model, once trained, reached a $AP_{IoU\geq 0.5}$ of 0.937, and a $AR_{IoU\geq 0.5}$ of 0.959.

All the approaches presented in this paper were found to be efficient ways of solving the building segmentation task proposed on satellite images. Interestingly, each approach came with its own adaptations, and the scores attained by the different proposed models reached similar $AP_{IoU\geq 0.5}$ and $AR_{IoU\geq 0.5}$ scores.

## Data Availability

All the datasets used in the experiments in the paper are available at: https://www.aicrowd.com/challenges/mapping-challenge. The test dataset is not released, as the goal of this work is to set up an ongoing benchmark. However, participants can evaluate their models by submitting solutions to the page here: https://www.aicrowd.com/challenges/mapping-challenge.

## Footnotes

a The individual training, validation and test sets are available at: https://www.aicrowd.com/challenges/mapping-challenge/dataset_files

b open source framework, available here: https://pytorch.org/
